# Prognostic Value of an Immune-Related Gene Signature in Oral Squamous Cell Carcinoma

**DOI:** 10.3389/fonc.2021.776979

**Published:** 2021-12-21

**Authors:** Chao Zhu, Liqun Gu, Mianfeng Yao, Jiang Li, Changyun Fang

**Affiliations:** ^1^ Department of Stomatology, Xiangya Hospital, Central South University, Changsha, China; ^2^ Department of Pediatric Stomatology, Xiangya Stomatological Hospital, Central South University, Changsha, China

**Keywords:** immune-related genes, oral squamous cell carcinoma, prognosis, tumor microenvironment, immunotherapy

## Abstract

The prognosis and immunotherapy response rates are unfavorable in patients with oral squamous cell carcinoma (OSCC). The tumor microenvironment is associated with tumor prognosis and progression, and the underlying mechanisms remain unclear. We obtained differentially expressed immune-related genes from OSCC mRNA data in The Cancer Genome Atlas (TCGA) database. Overall survival-related risk signature was constructed by univariate Cox regression analysis and LASSO Cox regression analysis. The prognostic performance was validated with receiver operating characteristic (ROC) analysis and Kaplan–Meier survival curves in the TCGA and Gene Expression Omnibus (GEO) datasets. The risk score was confirmed to be an independent prognostic factor and a nomogram was built to quantify the risk of outcome for each patient. Furthermore, a negative correlation was observed between the risk score and the infiltration rate of immune cells, as well as the expression of immunostimulatory and immunosuppressive molecules. Functional enrichment analysis between different risk score subtypes detected multiple immune-related biological processes, metabolic pathways, and cancer-related pathways. Thus, the immune-related gene signature can predict overall survival and contribute to the personalized management of OSCC patients.

## Introduction

Oral squamous cell carcinoma (OSCC) is one of the common malignant neoplasms in the head and neck region (1), leading to approximately 1.8% cancer-related death worldwide in 2020 (2). In the United States, there are an estimated 35,540 new cases and 6,980 deaths in 2021. In spite of the advantages of multimodal therapy including surgical resection, with or without radiotherapy or chemotherapy, the 5-year survival rate is approximately 50% (3). The challenge highlights the need to identify prognostic biomarkers to predict survival in patients with OSCC.

Over the past decade, immunotherapy has proven to be an effective treatment for various cancers. The identification of possible mechanisms of immune evasion has improved the understanding of cancer immunotherapy (4). Cancer immunotherapy, particularly immune checkpoint inhibitors (ICIs), has shown durable anti-tumor activity and improved survival in patients with head and neck squamous cell carcinoma (HNSCC) (5). Despite initial enthusiasm, only a small number of patients have benefited from immunotherapy (6, 7). The complex interactions between cancer and the immune system have elucidated the role of the immune system in cancer development. To estimate the potential response to ICIs treatment, further exploration of predictive biomarkers is necessary.

In this study, we aimed to assess the correlation between immune-related genes and the prognosis and immune landscape of OSCC. Finally, we further performed functional enrichment analysis to explore the underlying mechanisms.

## Materials and Methods

### Data Sources

RNA sequencing and clinical data of 325 OSCC and 32 normal oral cavity samples in The Cancer Genome Atlas (TCGA) database were obtained from the UCSC Xena data portal^1^ and eBioPortal^2^ database. The GSE41613 and GSE42743 were obtained from the Gene Expression Omnibus (GEO) database^3^ (8). The gene expression data of the GEO database were normalized by rma method using affy R package (9).

### Construction of Risk Score Model

To identify differentially expressed genes (DEGs) between normal and tumor samples in the TCGA dataset, RNA sequencing data were performed using the limma R package with a cutoff of |log_2_FC| ≥ 1.5 and a false discovery rate (FDR) < 0.05 (10). We extracted immune-related DEGs from the identified DEGs based on the ImmPort database^4^ (11). Univariate Cox regression analysis was used to estimate the association between the expression of immune-related DEGs and overall survival (OS) of patients. Next, the LASSO regression model was conducted to identify key prognostic genes using the glmnet R package (12). Risk scores for each OSCC sample were derived based on the expression of prognostic genes and their corresponding regression coefficient.

### Internal and External Validation of the Prognostic Signature

Patients in the TCGA dataset were randomly divided into a training set (*n* = 162) and a testing set (*n* = 163) for internal validation. The GSE41613 and GSE42743 datasets were used as the external validation cohort. Overall survival (OS), disease-specific survival (DSS), and progression-free survival (PFS) were plotted using Kaplan–Meier curves and calculated using Cox regression analysis. Patients were divided into high-risk and low-risk groups based on the median value of the risk score. Time-dependent receiver operating characteristic (ROC) curve was performed to assess the predictive efficiency of the prognostic signature using the timeROC R package (13). Independent prognostic factors were identified by multivariate Cox regression analysis using the survival R package (14). Furthermore, all independent prognostic factors obtained by multivariate Cox regression were used to construct a predictive nomogram by the rms R package to assess the 1-year, 3-year, and 5-year OS of the patients. Its predictive capacities were estimated by the corresponding calibration curve and the consistency index (C Index). Then, decision curve analysis (DCA) was performed by the dcurver R package to investigate the clinical utility of the nomogram model.

### Estimation of the Immune Landscape

We estimated the expressions of 782 genes from 28 types of immune cells to quantify the infiltration ratio of immune cells (15). The ratio of immune cell infiltration was calculated by the ssGSEA method through the Gene Set Variation Analysis (GSVA) R package and visualized by heatmap R package (16, 17). The stromal, immune, and estimate scores were quantified by the estimate R package (18). Data on stromal fraction, leukocyte fraction, scores of six representative signatures, and the gene set of immune-related markers were obtained from a previously published study from the TCGA group (19).

### Functional Enrichment Analysis

Functional enrichment analysis of Gene Ontology (GO) terms, Kyoto Encyclopedia of Genes and Genomes (KEGG) pathways, and Hallmark pathways was analyzed using the GSEA software v4.1.0 and visualized by ggplot2 R package (20, 21).

### Statistical Analysis

Data comparison between two groups was performed by two-tailed *t*-test and multiple *t*-tests with FDR < 0.05 for continuous comparisons. Data comparison between three groups was performed by one-way ANOVA test. Correlations between ssGSEA scores of 28 immune cells and risk scores or the expression of the prognostic signature were determined by Pearson correlation test. In all analyses, *p* < 0.05 was considered statistically significant. All statistical analyses were conducted by GraphPad Prism v8.0.2 and R software v4.0.5.

## Results

### Identification of the Candidate Immune-Related Genes

Differential expression analysis was performed between normal and tumor samples. A total of 1,313 upregulated genes and 1,615 downregulated genes were identified (**Figure S1A**). By comparing the DEGs and immunologically relevant genes, 249 genes overlapped (**Figure S1B**), and the expression of these genes was shown in the heatmap (**Figure S1C**). Univariate Cox regression analysis was performed to explore the correlation between the expression of 249 immune-related DEGs and OS in patients with OSCC. In total, 16 candidate immune-related genes were identified (**Figure S1D**).

### Construction and Internal Validation of the Prognostic Signature

**Figure 1:**
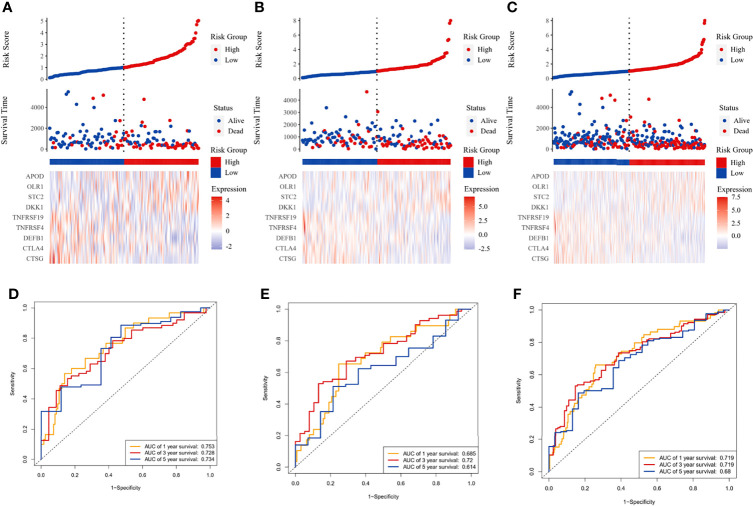
Immune-related prognostic model construction in the TCGA cohort. The prognostic significance of risk scores was evaluated using the training set **(A, D)**, the testing set **(B, E)**, and the whole TCGA set **(C, F)**, respectively. **(A–C)** The first panel from top represents the risk score distribution of the samples. The intersecting point represents the median of risk scores. The second panel from the top was the distribution of OS status and risk scores. The bottom panel was the heatmap of the mRNA expression of the nine immune-related DEGs. **(D–F)** The ROC curve for predicting 1-, 3-, and 5-year overall survival probability.

**Figure 2:**
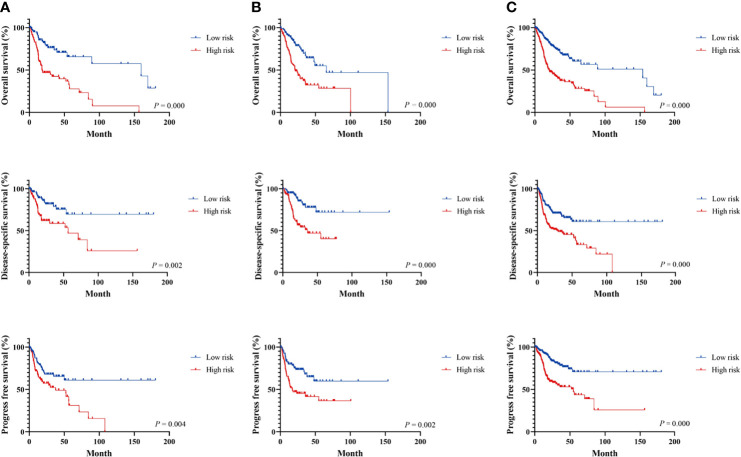
Survival analysis of immune-related signature in the TCGA cohort. Kaplan–Meier curves for the survival rate of OSCC patients between the high-risk and low-risk groups in the training **(A)**, testing **(B)**, and whole TCGA set **(C)**, respectively. *p*-values for significance (<0.05) calculated using Cox regression analysis.

The LASSO Cox regression analysis was used to further identify 9 key genes, namely, Apolipoprotein D (APOD), Oxidized Low Density Lipoprotein Receptor 1(OLR1), Stanniocalcin-2 (STC2), Dickkopf-related protein 1 (DKK1), Tumor necrosis factor receptor superfamily member 19 (TNFRSF19), tumor necrosis factor receptor superfamily member 4 (TNFRSF4), Defensin Beta 1(DEFB1), Cytotoxic T-Lymphocyte Associated Protein 4 (CTLA4), and Cathepsin G (CTSG) (**Figures S1E, F**). Risk scores were calculated according to the expression of these prognostic genes weighted by the coefficients in the regression analysis for each OSCC sample. Patients from the training set, the testing test, and the entire TCGA set were divided into high-risk and low-risk groups based on the median value of the risk score, respectively. A higher proportion of deaths was observed in the high-risk group than that in the low-risk group (first and second panel of **Figures 1A–C**). The 9 genes were differentially expressed between the high-risk and low-risk groups (bottom panel of **Figures 1A–C** and **Figure S2**). To assess the predictive performance of the 9-gene prognostic signature, time-dependent ROC analyses were performed in the training, testing, and whole TCGA set to estimate the 1-year, 3-year, and 5-year OS probability (**Figures 1D–F**). Patients with low-risk scores showed longer OS, DSS, and PFS in the training, testing, and whole TCGA set (**Figures 2A–C**). We also found higher proliferation scores and wound healing scores in the high-risk group (**Figures S3A, B**). Together, these supported the predictive ability of the prognostic signature.

### External Validation of the Prognostic Model in the GEO Cohort

**Figure 3:**
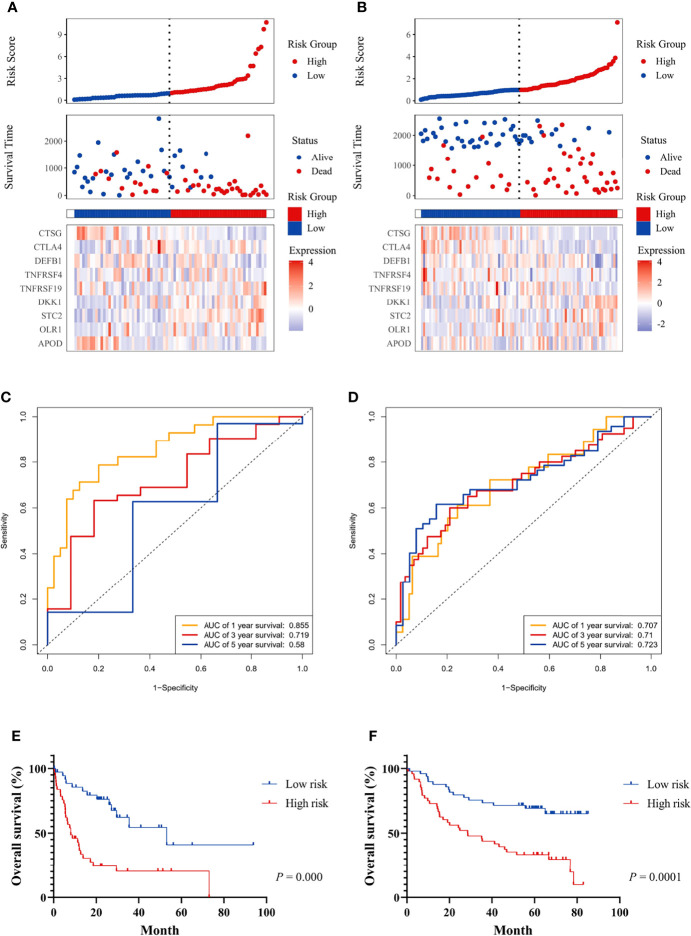
The prognostic significance of the risk score and survival analysis were evaluated using the GEO validation cohort. The prognostic significance of risk scores was evaluated using the validation datasets GSE42743 **(A)** and GSE41613 **(B)**, respectively. The first from top represents the risk scores distribution of the samples. The intersecting point represents the median of risk scores. The second from top was the distribution of OS status and risk scores. The bottom panel was the heatmap of the mRNA expression of the nine immune-related DEGs. The ROC curve for predicting 1-, 3-, and 5-year overall survival probability in GSE42743 **(C)** and GSE41613 **(D)**. Kaplan–Meier curves for the survival rate of OSCC patients between the high-risk and low-risk groups in GSE42743 **(E)** and GSE41613 **(F)**. *P* values for significance (<0.05) calculated using Cox regression analysis.

Patients in the GEO datasets were divided into high-risk and low-risk groups by the median value of risk scores. The high-risk group had a higher proportion of deaths compared to the low-risk group (**Figures 3A, B**). The ROC analysis verified the predictive efficiency of estimating the 1-year, 3-year, and 5-year OS probability (**Figures 3C, D**). The patients in the high-risk group had a worse prognosis (**Figures 3E, F**).

### The Risk Score is an Independent Prognostic Factor and Its Relationship to Clinical Characteristics

**Figure 4:**
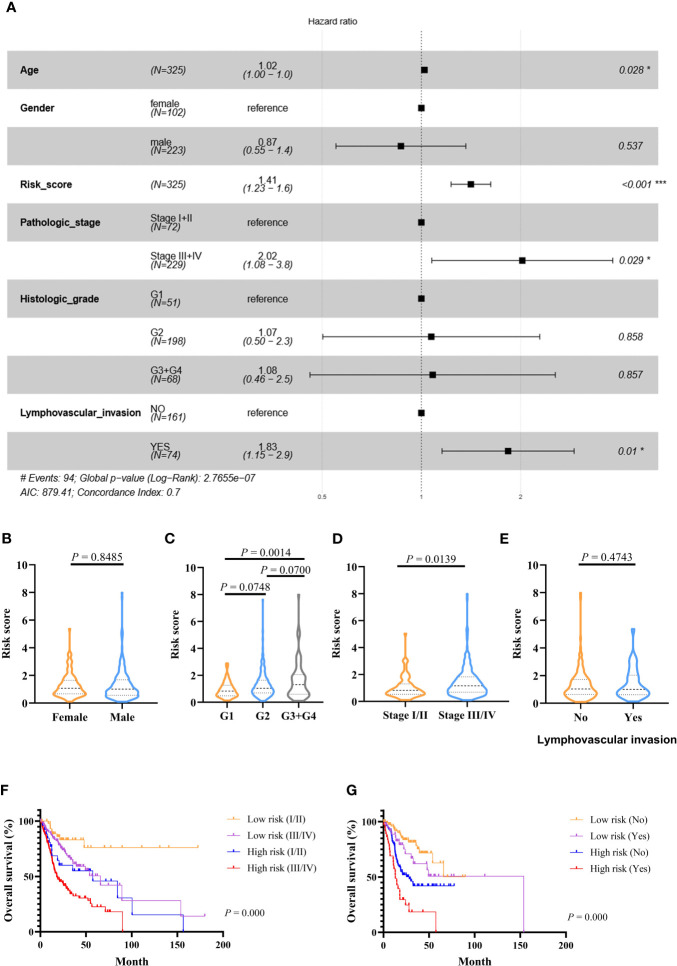
Prognostic values of the immune-related signature model in the TCGA cohort. **(A)** Multivariate Cox regression analysis regarding OS in OSCC. **(B–E)** The distribution of risk scores in OSCC samples stratified by gender, histologic stage, pathologic stage, and lymphovascular invasion. **(F)** Kaplan–Meier curves for patients stratified by both pathologic stage and risk scores. **(G)** Kaplan–Meier curves for patients stratified by both lymphovascular invasion and risk scores. *p* < 0.05 shows significant difference. Survival significance calculated using Cox regression analysis. # just indicates the Events number. *p value < 0.05, **p value < 0.01, ***p value < .001.

Multivariate Cox-regression analysis was performed using risk scores and clinical parameters as covariates to evaluate the independence of the risk score. The result demonstrated that the risk score can be considered as an independent predictor (TCGA: **Figure 4A**, GEO: **Figures S4A, B**). Then, we analyzed the correlation between the prognostic signature and clinical characteristics. In the TCGA cohort, the risk score was significantly different among different histologic stage and pathologic stage (**Figures 4C, D**). There were no differences between the risk score and age and gender (**Figures 4B, E**). In addition, OS was significantly shorter in high-risk patients with the same pathologic stage, and lymphovascular invasion status compared with low-risk patients (**Figures 4F, G**). In the GEO cohort, risk scores were higher in the stage III/IV group (**Figures S4C, D**), and the risk score could differentiate patients with the same pathological stage (**Figures S4E, F**).

### Development and Assessment of the Predictive Nomogram

**Figure 5:**
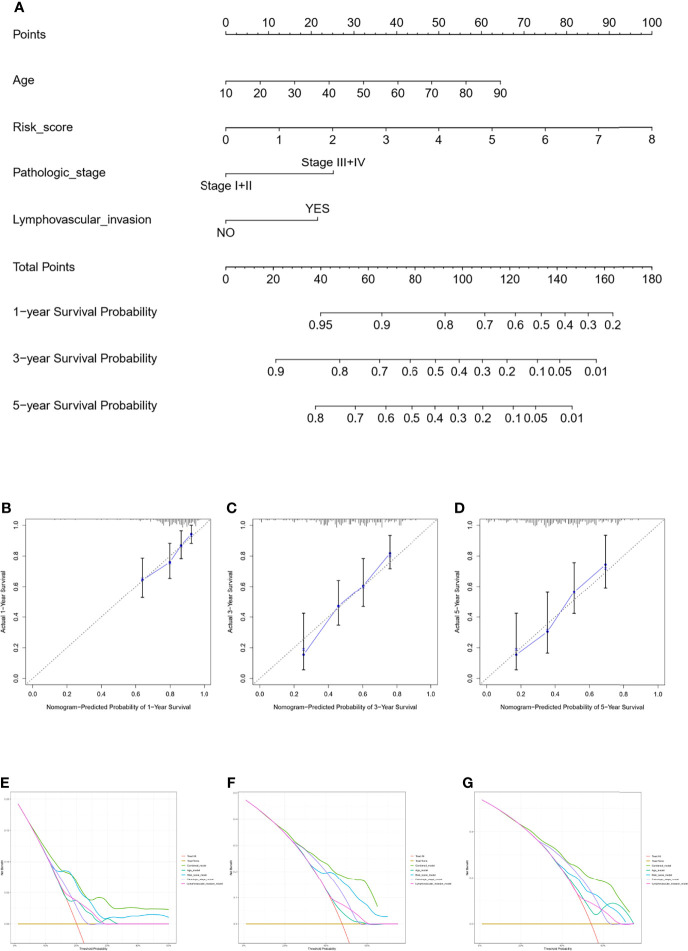
Nomogram for predicting the survival probability of OSCC patients in the TCGA cohort. The nomogram for prediction of the 1-, 3-, and 5-year survival probability for OSCC patients **(A)**. The calibration curve for prediction of the 1-year **(B)**, 3-year **(C)**, and 5-year **(D)** survival probability for OSCC patients. The DCA curves of the age, risk score, pathologic stage, lymphovascular invasion, and combined nomogram model compared for 1-year **(E)**, 3-year **(F)**, and 5-year **(G)** OS of OSCC.

The nomogram model was constructed using the independent factors including age, risk scores, pathologic stage, and lymphovascular invasion status in the TCGA dataset (**Figure 5A**). The calibration curve was close to the standard curve showing the accuracy of the predictive nomogram in predicting the probability of OS over 1, 3, and 5 years (**Figures 5B–D**). Then, we performed a decision curve analysis (DCA) for age, risk scores, pathologic stage, lymphovascular invasion status, and combined nomogram model to evaluate the clinical utility of the nomogram (**Figures 5E–G**).

### Correlation Between Tumor Immune Microenvironment and the Prognostic Signature

**Figure 6:**
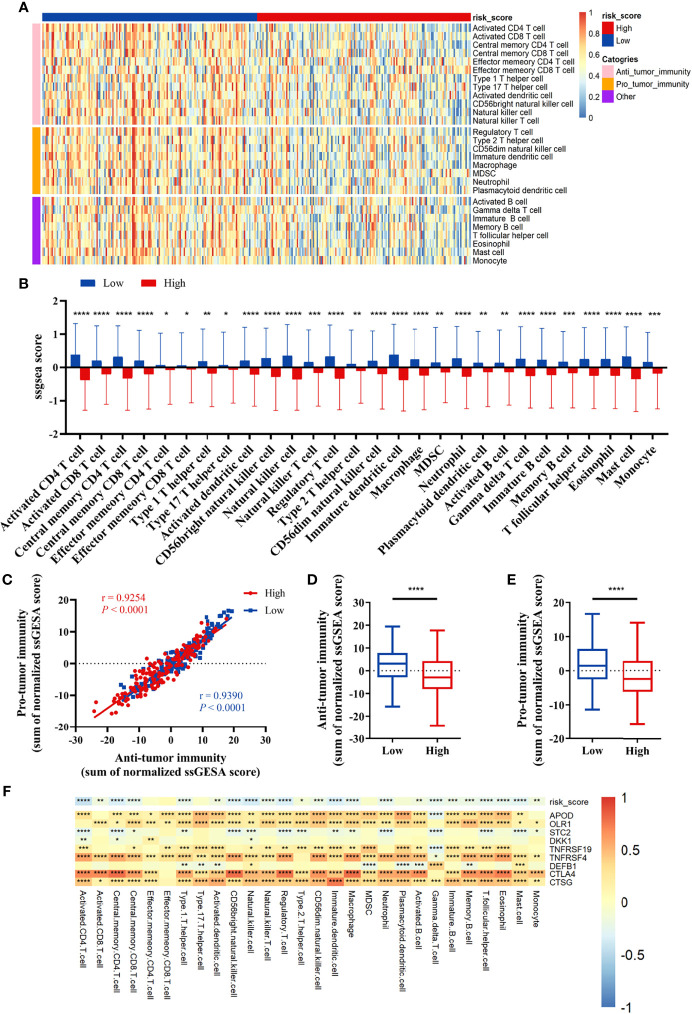
Correlation between immune cell infiltration and the prognostic signature in the TCGA cohort. **(A, B)** The infiltration ratio of 28 immune cells. **(C)** Correlation of the cells with anti-tumor immunity and pro-tumor immunity. **(D, E)** Anti-tumor immunity and pro-tumor immunity scores of the risk score model. **(F)** The correlation between the immune-related signature and the ssGSEA scores of 28 immune cells. All *p*-values for significance (<0.05) represent comparisons *via* two-tailed *t*-test and multiple *t*-tests with FDR < 0.05. *p-value < 0.05, **p-value < 0.01, ***p-value < 0.001, and ****p-value < 0.0001.

**Figure 7:**
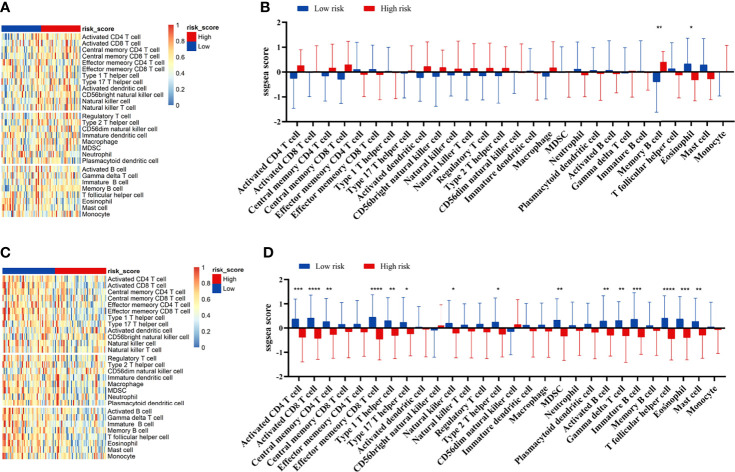
Correlation between immune cell infiltration and the prognostic signature in the GEO cohort. The infiltration ratio of 28 immune cells in GEO42743 **(A, B)** and GEO41613 **(C, D)**. All *p*-values for significance (<0.05) represent comparisons *via* two-tailed multiple *t*-tests with FDR < 0.05. *p-value < 0.05, **p-value < 0.01, ***p-value < 0.001, and ****p-value < 0.0001.

**Figure 8:**
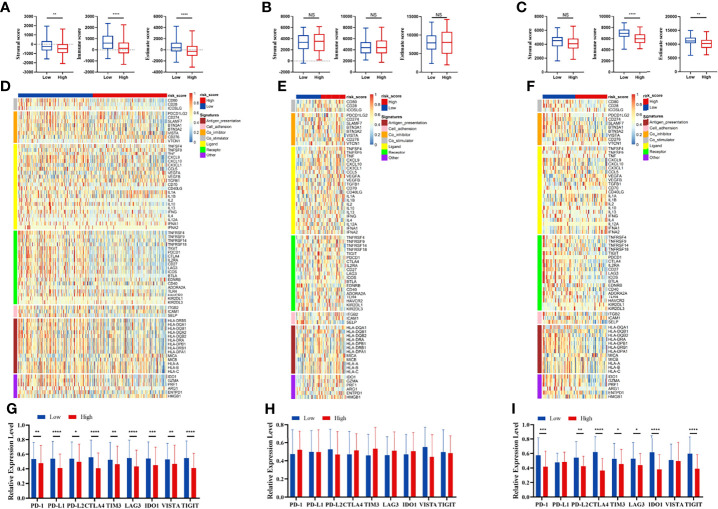
Immune patterns of the risk score model. Comparison of stromal scores, immune scores, and estimate scores between the high-risk and low-risk patients in the TCGA **(A)**, GSE42743 **(B)**, and GSE41613 **(C)**. The expression level of immune-related signatures in the TCGA **(D)**, GSE42743 **(E)** and GSE41613 **(F)**. The expression level of immune checkpoint molecules in the TCGA **(G)**, GSE42743 **(H)** and GSE41613 **(I)**. All *p*-values for significance (<0.05) represent comparisons *via* two-tailed *t*-test and multiple *t*-tests with FDR < 0.05. *p-value < 0.05, **p-value < 0.01, ***p-value < 0.001, ****p-value < 0.0001, and NS (not significant).

We compared the infiltration ratio of 28 immune cells. The high-risk group showed a relatively lower ratio of immune cell infiltration, including cells with anti-tumor activity and immunosuppressive activity (TCGA: **Figures 6A, B**; GEO: **Figure 7**). In addition, a positive correlation was observed between the ssgsea score of these two categories of immune cells in the high-risk and low-risk groups (**Figure 6C**). We compared the infiltration ratio of these two categories of immune cells in different risk groups and observed that the low-risk group was characterized by higher anti-tumor and pro-tumor immunity (**Figures 6D, E**). The risk score was negatively correlated with the enrichment score for most types of immune cells. The expression of CTSG, CTLA4, TNFRSF4, APOD, and OLR1 was positively correlated with the enrichment score of most immune cells, and the expression of STC2 was negatively correlated with it (**Figure 6F**). Using the ESTIMATE database, we observed higher stromal scores, immune scores, and estimate scores in the low-risk group (**Figures 8A–C**). We compared the stromal fraction and leukocyte fraction of these two groups in the TCGA cohort. The results showed that the stromal fraction and leukocyte fraction were higher in the low-risk group (**Figures S3C, D**). In addition, scores of macrophage regulation, lymphocyte infiltration and IFN-γ response were higher in the low-risk group in the TCGA cohort (**Figures S3E–G**). While, scores of homologous recombination defects were lower in the low-risk group and no differences were found in TGF-β response (**Figures S3H, I**). After analyzing the expression profiles of 75 immune-related genes in different risk groups, it was observed that the expression of immune-stimulatory and suppressive genes was relatively higher in the low-risk group (**Figures 8D–F**). When comparing the expression levels of several important inhibitory checkpoint molecules between the high-risk and low-risk groups, we found that the expression levels of Programmed cell death protein 1 (PD-1), Programmed death-ligand 2 (PD-L2), Cytotoxic T-Lymphocyte Associated Protein 4 (CTLA4), T-cell immunoglobulin 3 (TIM3), Lymphocyte activation gene 3 (LAG3), Indoleamine 2,3-dioxygenase 1 (IDO1), and T-cell immunoreceptor with immunoglobulin and immunoreceptor tyrosine-based inhibitory motif domains (TIGIT) were higher in the low-risk group (**Figures 8G–I**).

### Functional Enrichment Analysis

**Figure 9:**
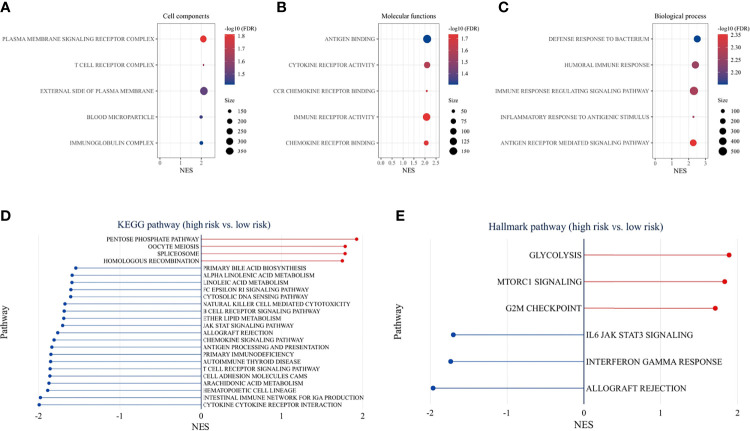
Functional enrichment analysis. GO pathway enrichment analysis revealed top 5 GO terms in cellular components **(A)**, molecular functions **(B)**, and biological process **(C)**. KEGG pathway analysis **(D)** and hallmark pathway analysis **(E)** between the high-risk and low-risk groups (*p* < 0.05 and FDR <25% were controlled).

GO enrichment analysis for different risk groups revealed the following top immune-related GO terms: T-cell receptor complex, plasma membrane signaling receptor complex, and immunoglobulin complex in cellular components (**Figure 9A**); antigen binding, cytokine receptor activity, and CCR chemokine receptor binding in molecular functions (**Figure 9B**); defense response to bacterium, humoral immune response, and immune response regulation signaling pathway in biological process (**Figure 9C**). KEGG pathway analysis showed that immune-related pathways and metabolic pathways were enriched in the low-risk group, while the pentose phosphate pathway (PPP), spliceosome pathway, and homologous recombination (HR) pathway were enriched in the high-risk group (**Figure 9D**). Furthermore, hallmark pathway analysis revealed that glycolysis, mammalian target of rapamycin complex 1 (mTORC1) signaling, and G2M checkpoint were enriched in the high-risk group, whereas IL6/Jak/Stat3 signaling, Interferon-γ response, and allograft rejection were enriched in the low-risk group (**Figure 9E**).

## Discussion

ICIs are effective in the treatment of multiple cancers and have greatly improved the outcomes of patients. The limitation is that only a small number of patients benefit from ICIs treatment, including HNSCC (6, 7). Immune cells are key regulatory components of the tumor microenvironment (TME) and play an important role in tumor growth and progression (22). Immune cell infiltration is associated with the survival rate of OSCC patients (23). However, the underlying mechanisms still need further elucidation.

In this study, we firstly identified immune-related genes that are differentially expressed between normal and tumor tissues. Then, univariate Cox regression analysis screened 16 survival-related genes. These survival-related genes have the potential to be biomarkers for prognosis. Furthermore, we established an immune-related risk signature, which is composed of 9 genes (APOD, OLR1, STC2, DKK1, TNFRSF19, TNFRSF4, DEFB1, CTLA4, and CTSG). APOD, OLR1, STC2, and DKK1 were overexpressed in high-risk patients. APOD has been reported to exhibit tumor suppressive activity in some types of tumors (24). OLR1, STC2, and DKK1 correlate with tumor evolution and immunosuppressive effects (25–29). TNFRSF19, TNFRSF4, DEFB1, CTLA4, and CTSG were identified as protective genes. High expression of TNFRSF19 is associated with poor prognosis in various types of cancer (30, 31). TNFRSF4, a T-cell co-stimulatory molecule, enhances CD8^+^ T-cell infiltration (32). DEFB1 suppresses tumor migration and invasion in OSCC (33). CTLA-4 is a negative regulator of T-cell activation, and CTLA-4 inhibitors have been shown to promote antitumor immunity (34). CTSG is regarded as an immune-related biomarker in OSCC and inhibits OSCC cell proliferation, migration, and invasion (35). The specific role of the immune-related genes needs further investigation.

The immune-related signature could be used as an independent predictor of the prognosis in the TCGA cohort and GEO cohort. The signature could divide OSCC patients into high-risk and low-risk groups with statistically different survival outcomes. The higher proliferation score and wound healing score in the high-risk group could partially explain the worse prognosis of patients with high-risk scores. In addition, the risk score could stratify patients with the same pathological stage, and lymphovascular invasion status. Additionally, the nomogram model further demonstrated that the risk signature can predict long-term prognosis. To assess the clinical utility of our signature, the DCA curve revealed that the nomogram joined the risk score, and clinical factors have a higher predictive efficiency than a single clinical factor. These data suggest that this immune-related risk signature can predict the prognosis of OSCC patients.

Immune cell infiltration has been reported to be an important indicator of tumor prognosis. Immune scores, as well as scores for macrophages, lymphocyte infiltration and IFN-γ response were higher in the low-risk group. These indicate a complex intratumoral immune state. Then, we analyzed the immune cell infiltration and immune-related signatures of the high-risk and low-risk groups. The risk score was negatively correlated with the infiltration ratio of immune cells, suggesting that tumor cell infiltration is indicative of better prognosis. The low-risk group had a higher proportion of anti-tumor immune cells, including activated CD4^+^ T cells, activated CD8^+^ T cells, and natural killer (NK) cells. In addition, we also found higher levels of immunosuppressive cells, such as Treg cells, macrophages, and myeloid-derived suppressor cells (MDSCs) in the low-risk group. CD8^+^ T cells and NK cells, representing an activated phenotype, were higher expressed in the low-risk group, and correlated with better survival in HNSCC (36). These indicate that both anti-tumor immune cells and immunosuppressive cells are infiltrated in the TME in the low-risk group. Together, these findings suggest that the low-risk group is of the “hot tumor” phenotype, while the high-risk group is of the “cold tumor” phenotype, which could explain the difference in survival rates (37).

Consistent with immune cell infiltration phenotype, immune stimulatory factors and immune inhibitory factors were both higher expressed in the low-risk group. Co-expression of inhibitory factors had been observed following the infiltration of T cells (37, 38). The expression of negative regulatory immune checkpoints, including PD-1, PD-L2, CTLA-4, TIM3, LAG3, IDO1, and TIGIT, was relatively higher expressed in the low-risk group. The infiltration of immunosuppressive cells and elevated inhibitory pathways in the low-risk group may be negative feedback of anti-tumor immune activation. Collectively, these findings suggest that the low-risk group may be more sensitive to ICIs treatment.

To understand the mechanisms underlying the signature, functional enrichment analysis was performed between risk groups. GO analysis detected that immune-related GO terms were enriched in the low-risk group. KEGG pathway analysis showed that immune-related pathways and metabolic pathways were enriched in the low-risk group, while the PPP, spliceosome pathway, and HR pathway were enriched in the high-risk group. Further analysis of hallmark pathways revealed that glycolysis, mammalian target of rapamycin complex 1 (mTORC1) signaling, and G2/M checkpoint were enriched in the high-risk group, whereas IL6/Jak/Stat3 signaling, Interferon-γ response, and allograft rejection were enriched in the low-risk group. Recent studies have shown that IFN-γ upregulates immunosuppressive molecules such as PD-L1, PD-L2 and IDO1, in cancer and host cells (38, 39), thereby increasing the response likelihood to ICIs therapy. Cell metabolism is crucial for tumor immunity (40). On the one hand, fatty acids are required for anti-tumor effects, including the development and effector functions of CD8^+^ T cells (41). However, it was also found that fatty acids are important for Treg survival and function (42). Fatty acid metabolism can modulate the TME, and the adaptation of immune metabolism may partly explain the immune cell infiltration and expression of immune-related genes in the TME. In the high-risk group, cancer-related pathways were activated, which promoted the malignant transformation of the tumor and indicated a poor prognosis. Increased glycolytic activity in high-risk patients may lead to glucose competition within the TME, thereby limiting T-cell proliferation and effector functions (43). The PPP is another important metabolic pathway that helps cancer cells to meet anabolic requirements for nucleic acid synthesis, nicotinamide-adenine dinucleotide phosphate (NADPH) production, fatty acid synthesis and cell survival, as well as scavenging oxidative stress (44). Activation of mammalian target of rapamycin complex 1 (mTORC1) has been reported to stimulate PPP (45). An emerging role of spliceosome in cancer and immunity has been studied. Aberrant splicing contributes to cancer progression and immune dysregulation (46, 47). Spliceosome inhibitors have exhibited antitumor effects in cancer cells (48). The HR pathway is essential for DNA double-strand break (DSB) repair. Activation of HR in the high-risk group represented the onset of DNA damage. Higher HR deficits were found in the high-risk group, suggesting sensitivity to targeted therapy with poly ADP-ribose polymerase inhibitors (PARPi) (49) and DNA-damaging reagents (50). G2/M checkpoint was activated in the high-risk group in response to DNA damage. Small molecules targeting the G2/M checkpoint have shown promising results in preclinical studies (51). In summary, the low-risk group is the immune flamed phenotype and may potentially benefit from ICIs treatment, while targeting metabolic pathways, DNA damage or repair, and spliceosome may improve outcomes in the high-risk group.

The limitation is that the study is based on data available online. Further prospective studies with larger samples are needed to assess the clinical relevance of this signature, as well as *in vitro* and *in vivo* experimental studies to estimate its biological function in OSCC.

## Conclusion

In summary, we have established an immune-related prognostic signature that can predict the prognosis of patients with OSCC and potentially identify patients who may benefit from immunotherapy and therapies targeting metabolic pathways, DNA damage or repair, and spliceosome. These findings may provide insights into the precise management of OSCC.

## Data Availability Statement

The datasets presented in this study can be found in online repositories. The names of the repository/repositories and accession number(s) can be found at: UCSC Xena data portal (https://xenabrowser.net), eBioPortal (https://www.cbioportal.org), GSE41613 and GSE42743 from GEO database (https://www.ncbi.nlm.nih.gov/geo/), and ImmPort database (https://www.immport.org).

## Ethics Statement

Ethical review and approval was not required for the study on human participants in accordance with the local legislation and institutional requirements. Written informed consent for participation was not required for this study in accordance with the national legislation and the institutional requirements.

## Author Contributions

CZ designed this study and analyzed the data. CZ and LG carried out data acquisition. JL and MY helped interpret the data and prepared all figures. CZ, LG, JL, and CF wrote the manuscript. All authors contributed to the article and approved the submitted version.

## Funding

This work was supported by the National Natural Science Foundation of China (Grant No. 82071129) and the China Scholarship Council (Grant No. 201906370170 and No. 202106375007).

## Conflict of Interest

The authors declare that the research was conducted in the absence of any commercial or financial relationships that could be construed as a potential conflict of interest.

## Publisher’s Note

All claims expressed in this article are solely those of the authors and do not necessarily represent those of their affiliated organizations, or those of the publisher, the editors and the reviewers. Any product that may be evaluated in this article, or claim that may be made by its manufacturer, is not guaranteed or endorsed by the publisher.

## Footnotes

1
https://xenabrowser.net

2
https://www.cbioportal.org

3
https://www.ncbi.nlm.nih.gov/geo/

4
https://www.immport.org
