# Supplementary material for: Prognostic Value of an Immune-Related Gene Signature in Oral Squamous Cell Carcinoma
Source: Front Oncol. 2021 Dec 21;11:776979. doi: 10.3389/fonc.2021.776979 (PMC8724436; doi:10.3389/fonc.2021.776979)
Supplement: Supplementary file 1 [file DataSheet_1.docx]

**Supplementary Figure 1.** Identification of the candidate immune-related genes in the TCGA cohort. (**A**) Volcanic diagram of DEGs between normal and tumor samples. (**B**) Venn diagram to identify immune-related DEGs. (**C**) The expression profiles of immune-related DEGs between normal and tumor samples. (**D**) Univariate Cox regression analysis between the expressions of immune-related DEGs and overall survival (OS). (**E**) The LASSO coefficient profiles of survival-related immune-related DEGs. (**F**) Tenfold cross-validation for tuning parameter (lambda) selection in the LASSO model based on minimum criteria for OS. *P* < 0.05 shows significant difference.

**Supplementary Figure 2.** The expression level of the nine immune-related genes in the TCGA cohort. *P* values for significance (<0.05) represent comparisons via two-tailed t test. **P* value < 0.05, ***P* value < 0.01, ****P* value < 0.001, and *****P* value < 0.0001.

**Supplementary Figure 3.** Key immune characteristics in the TCGA cohort. *P* values for significance (<0.05) represent comparisons via two-tailed t test. **P* value < 0.05, ***P* value < 0.01, ****P* value < 0.001, *****P* value < 0.0001 and NS (not significant).

**Supplementary Figure 4.** Validation of the immune-related signature model in GEO cohorts. Multivariate Cox regression analysis regarding OS in OSCC in GEO42743 (A) and GEO41613 (B). The distribution of risk scores in OSCC samples stratified by tumor stage in GEO42743 (C) and GEO41613 (D). Kaplan-Meier curves for patients stratified by both tumor stage and risk scores in GEO42743 (E) and GEO41613 (F). P < 0.05 shows significant difference. Survival significance calculated using Cox regression analysis. **P* value < 0.05, ***P* value < 0.01, ****P* value < 0.001.


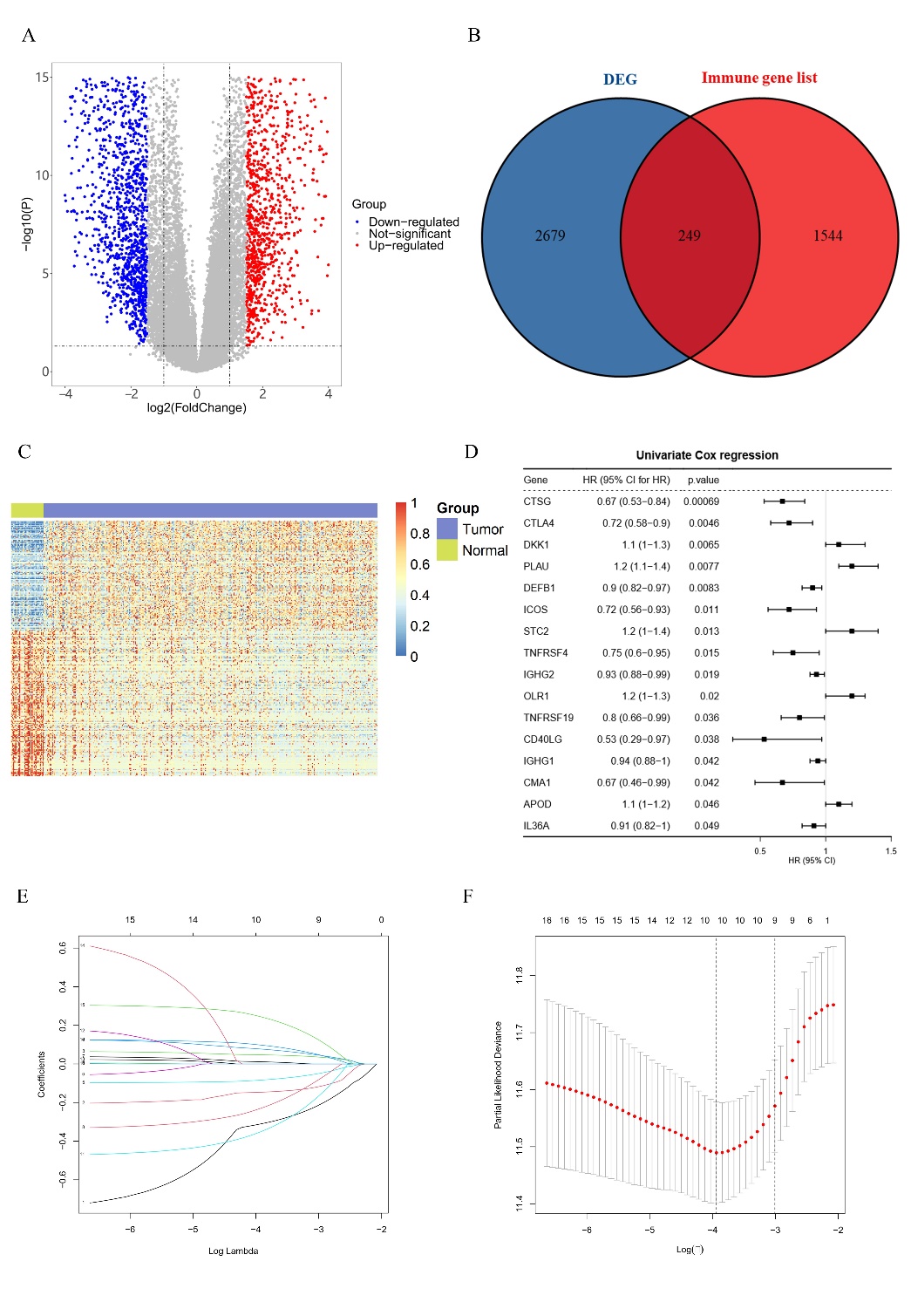


**Supplementary Figure 1.** Identification of the candidate immune-related genes in the TCGA cohort. (**A**) Volcanic diagram of DEGs between normal and tumor samples. (**B**) Venn diagram to identify immune-related DEGs. (**C**) The expression profiles of immune-related DEGs between normal and tumor samples. (**D**) Univariate Cox regression analysis between the expressions of immune-related DEGs and overall survival (OS). (**E**) The LASSO coefficient profiles of survival-related immune-related DEGs. (**F**) Tenfold cross-validation for tuning parameter (lambda) selection in the LASSO model based on minimum criteria for OS. *P* < 0.05 shows significant difference.

**
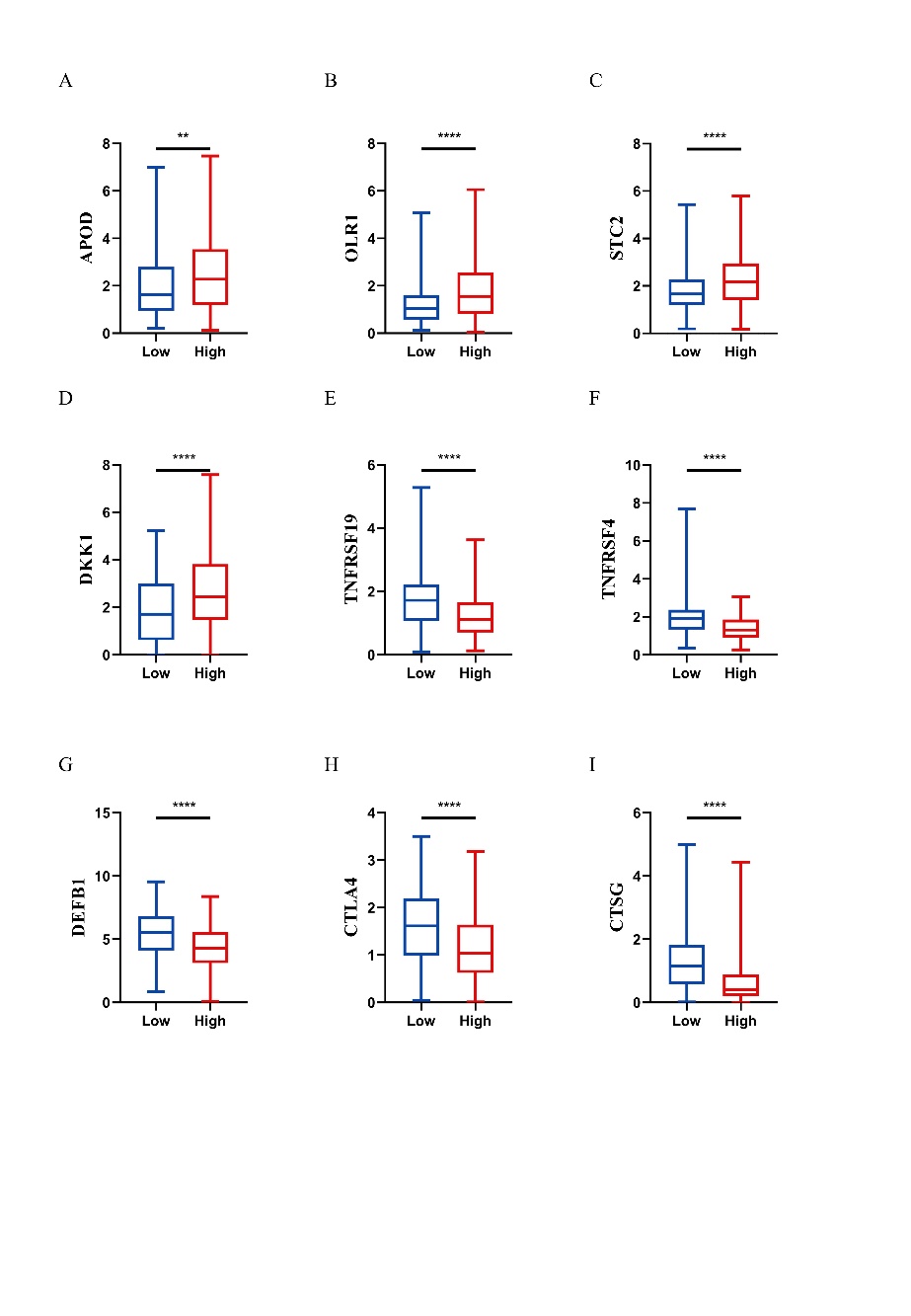
**

**Supplementary Figure 2.** The expression level of the nine immune-related genes in the TCGA cohort. *P* values for significance (<0.05) represent comparisons via two-tailed t test. **P* value < 0.05, ***P* value < 0.01, ****P* value < 0.001, and *****P* value < 0.0001.

**
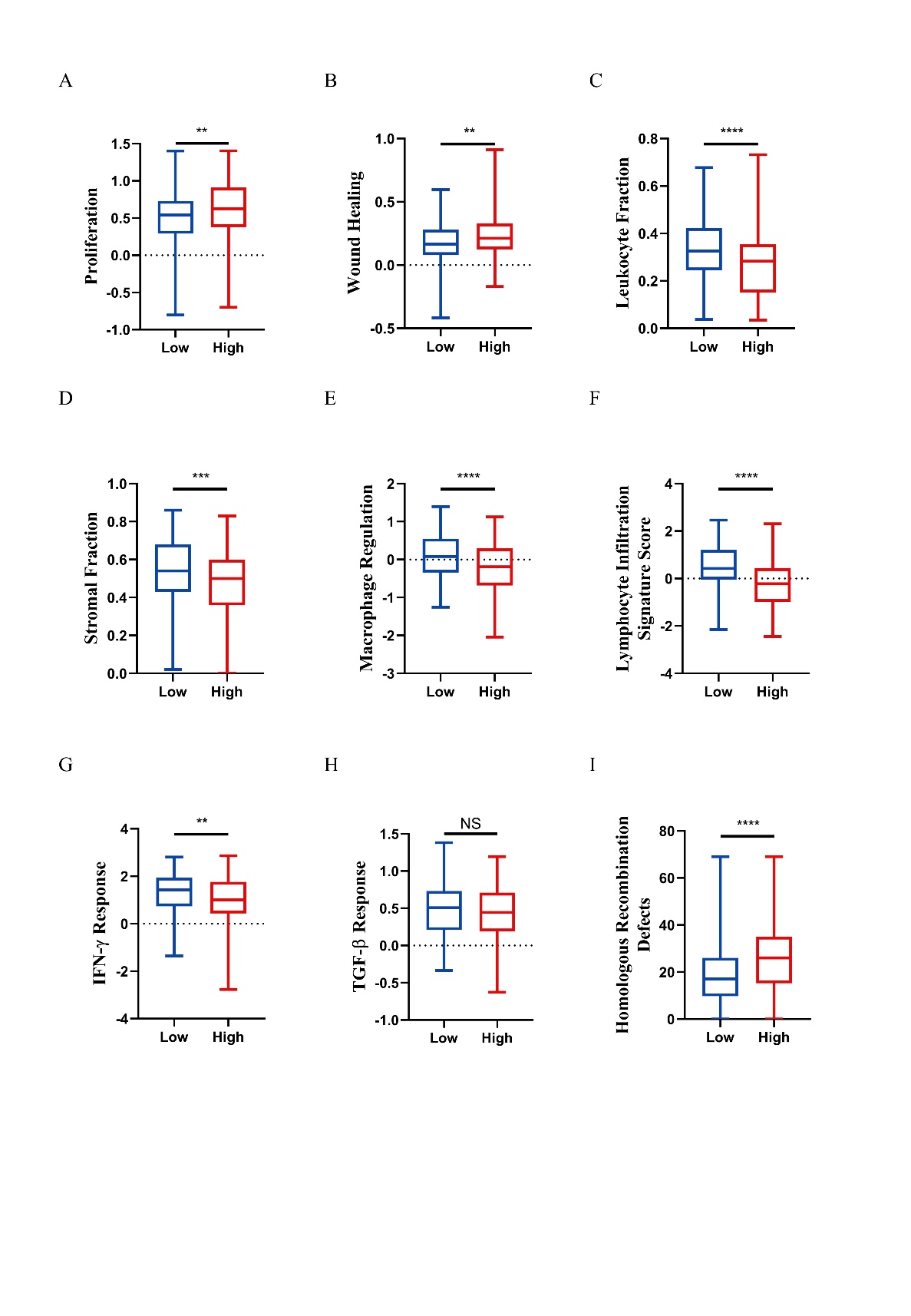
**

**Supplementary Figure 3.** Key immune characteristics in the TCGA cohort. *P* values for significance (<0.05) represent comparisons via two-tailed t test. **P* value < 0.05, ***P* value < 0.01, ****P* value < 0.001, *****P* value < 0.0001 and NS (not significant).


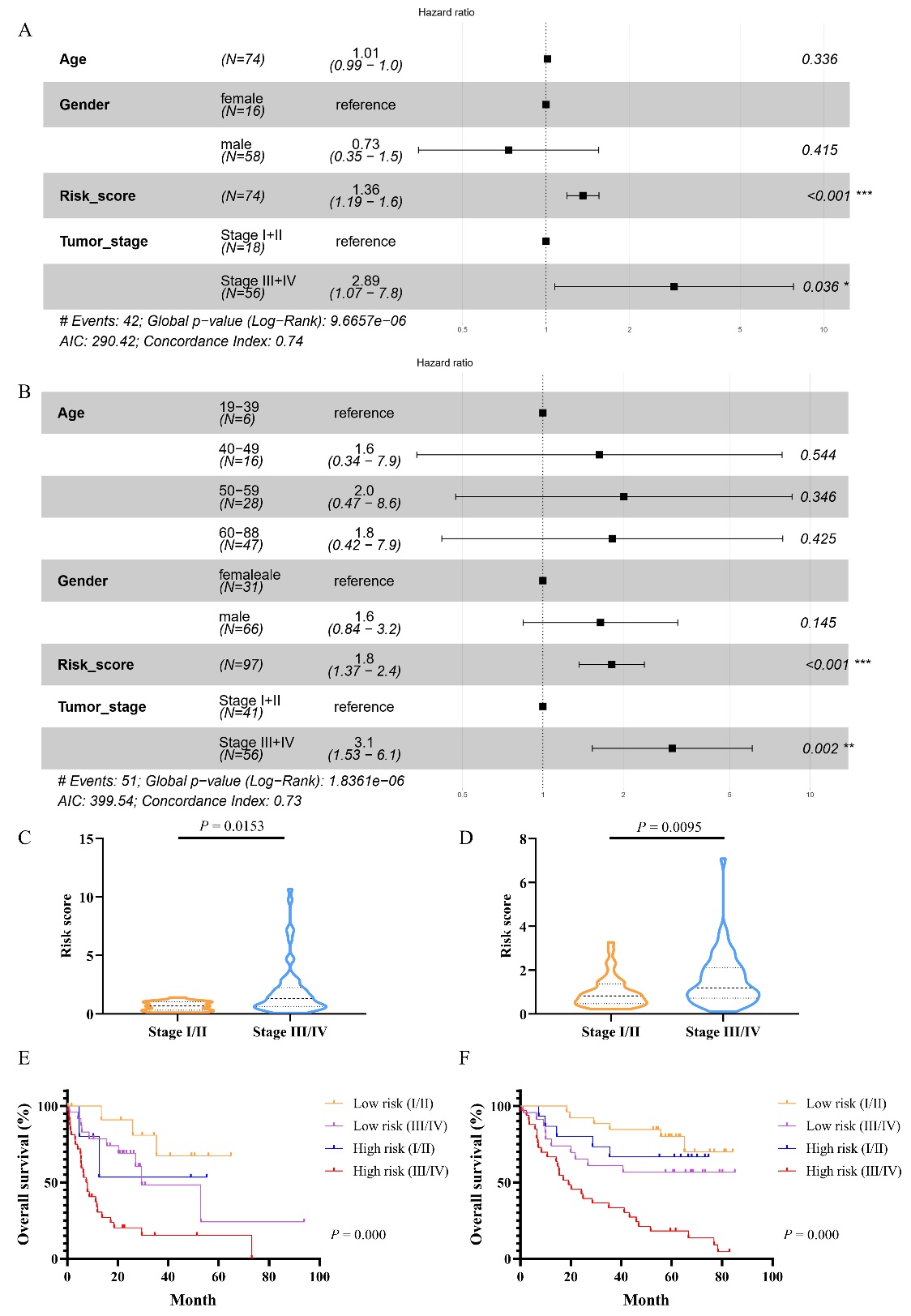


**Supplementary Figure 4.** Validation of the immune-related signature model in GEO cohorts. Multivariate Cox regression analysis regarding OS in OSCC in GEO42743 (A) and GEO41613 (B). The distribution of risk scores in OSCC samples stratified by tumor stage in GEO42743 (C) and GEO41613 (D). Kaplan-Meier curves for patients stratified by both tumor stage and risk scores in GEO42743 (E) and GEO41613 (F). P < 0.05 shows significant difference. Survival significance calculated using Cox regression analysis. **P* value < 0.05, ***P* value < 0.01, ****P* value < 0.001.
